# Genome-Wide Analysis Identifies Germ-Line Risk Factors Associated with Canine Mammary Tumours

**DOI:** 10.1371/journal.pgen.1006029

**Published:** 2016-05-09

**Authors:** Malin Melin, Patricio Rivera, Maja Arendt, Ingegerd Elvers, Eva Murén, Ulla Gustafson, Mike Starkey, Kaja Sverdrup Borge, Frode Lingaas, Jens Häggström, Sara Saellström, Henrik Rönnberg, Kerstin Lindblad-Toh

**Affiliations:** 1 Science for Life Laboratory, Department of Medical Biochemistry and Microbiology, Uppsala University, Uppsala, Sweden; 2 Science for Life Laboratory, Department of Immunology, genetics and pathology, Uppsala University, Uppsala, Sweden; 3 Evidensia Södra Djursjukhuset, Stockholm, Sweden; 4 Department of Veterinary Medicine, University of Cambridge, Cambridge, United Kingdom; 5 Broad Institute of MIT and Harvard, Cambridge, Massachusetts, United States of America; 6 Department of Animal Breeding and Genetics, Swedish University of Agricultural Sciences, Uppsala, Sweden; 7 Animal Health Trust, Newmarket, United Kingdom; 8 Department of Basic Sciences and Aquatic Medicine, Norwegian University of Life Sciences, Oslo, Norway; 9 Department of Clinical Sciences, Swedish University of Agricultural Sciences, Uppsala, Sweden; University of Bern, SWITZERLAND

## Abstract

Canine mammary tumours (CMT) are the most common neoplasia in unspayed female dogs. CMTs are suitable naturally occurring models for human breast cancer and share many characteristics, indicating that the genetic causes could also be shared. We have performed a genome-wide association study (GWAS) in English Springer Spaniel dogs and identified a genome-wide significant locus on chromosome 11 (p_raw_ = 5.6x10^-7^, p_perm_ = 0.019). The most associated haplotype spans a 446 kb region overlapping the *CDK5RAP2* gene. The CDK5RAP2 protein has a function in cell cycle regulation and could potentially have an impact on response to chemotherapy treatment. Two additional loci, both on chromosome 27, were nominally associated (p_raw_ = 1.97x10^-5^ and p_raw_ = 8.30x10^-6^). The three loci explain 28.1±10.0% of the phenotypic variation seen in the cohort, whereas the top ten associated regions account for 38.2±10.8% of the risk. Furthermore, the ten GWAS loci and regions with reduced genetic variability are significantly enriched for snoRNAs and tumour-associated antigen genes, suggesting a role for these genes in CMT development. We have identified several candidate genes associated with canine mammary tumours, including *CDK5RAP2*. Our findings enable further comparative studies to investigate the genes and pathways in human breast cancer patients.

## Author Summary

Dogs provide an excellent model system for several human diseases, including cancer. Heavy breeding for certain behavioural or phenotypic traits has created genetic isolates–breeds–characterised by low levels of genetic variation and a limited number of genetic disease variants within each breed. Cancer is the most common cause of death in dogs today, and canine mammary tumours (CMT) are the most prevalent tumour type in unspayed female dogs. These tumours are very similar to human breast cancer and could therefore be used as a naturally occurring model for the human disease. We have investigated genetic variants associated with CMT in English Springer Spaniels pointing to a gene involved in cell cycle regulation (*CDK5RAP2*). The *CDK5RAP2* could therefore have a key role in the development of mammary tumours and we suggest that further studies should be performed in both dogs and women to investigate *CDK5RAP2* and its possible effect on disease and treatment response.

## Introduction

Breast cancer is a devastating disease causing a majority of cancer-related deaths in women worldwide [1]. Sub-categorisation of patients based on receptor status (oestrogen (ER), progesterone (PR) and HER2) has enabled improved targeted treatments. However, treatment could be further improved, especially for the triple negative patients, which account for 12–24% of the patients, and for which no efficient therapy exists at present [2]. There is therefore an urgent need to identify predisposing genes and prognostic tools to improve early detection and enhanced treatment options in breast cancer. One approach is to attempt to identify genes influencing susceptibility to breast cancer, which also has the potential to reveal novel targets for drug development and assist in the implementation of strategies towards personalised medicine. Breast cancer susceptibility is generally believed to be conferred by a large number of loci, each contributing with a small effect to breast cancer risk [3]. So far only a small fraction of human breast cancer cases can be explained by a single gene mutation and the prevalence of clearly hereditary breast cancer is about 5–10% of all breast cancers, leaving a large majority of cases with a more complex aetiology [4,5]. Several genes predisposing to breast cancer have been identified, including *BRCA1* and *BRCA2*, which explain about 20% of the familial breast cancer cases [6]. A large number of association studies have been performed in search of breast cancer susceptibility genes, including pooled strategies and meta-analyses, and many genes conferring a moderately increased risk have been identified [7–10]. However, a large proportion of the inherited risk factors remain unknown.

The dog is a unique model for human disease, sharing many both complex and monogenic diseases, a similar gene set and largely the same environment as humans. In addition, the canine population structure makes trait mapping much easier than in humans. Several recent studies have proven the effectiveness of gene discovery in dog breeds for both monogenic [11–14] and complex traits including cancer [15–19]. Canine mammary tumours (CMT) are the most common neoplasia in intact female dogs and constitute about half of all tumours [20,21]. As in women, dogs develop mammary tumours with increasing age, rarely before 5 years of age and with a median age of occurrence of 10–11 years. However, the English Springer Spaniel (ESS) has been shown to have a median age of onset at 7 years of age in the Swedish dog population and 32% of the female dogs are affected at ten years of age in this high-risk breed [20]. This early onset mimics that of familial breast cancer in humans and indicates that inherited risk factors influence CMT development. CMTs also show a high degree of similarity to human breast tumours regarding epidemiological, clinical, morphological and prognostic features [22–24]. CMT is considered a heterogeneous disease with a complex background, resembling that of human breast cancer, but very little is known about the inherited genetic risk factors influencing CMT. We have previously shown that the *BRCA1*, *BRCA2* and *ESR1* genes are associated with CMT in Swedish ESS [25,26]. The associations imply similarities in predisposing genetic risk factors between human and canine mammary tumours, but explain only a minor proportion of the elevated risk for CMT in the ESS breed.

In this study, we have conducted a genome-wide association study (GWAS) to identify genetic risk factors associated with mammary tumour predisposition in the ESS breed in addition to *BRCA1/2* and *ESR1* [25,26]. We have identified three candidate regions containing plausible cancer susceptibility genes and pathways. The top associated region is located on canine chromosome 11 and reveals a complex genetic architecture with an abundance of risk haplotypes indicating involvement of the centrosomal cell cycle regulator CDK5 regulatory subunit-associated protein 2 (*CDK5RAP2*) in tumour development.

## Results

### GWAS identifies three regions significantly associated with CMT

**Fig 1:**
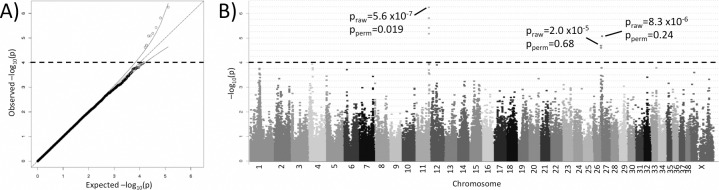
Genome-wide association results. **(A)** Quantile-quantile plot displaying a lambda of 1.00, indicating no residual inflation. Thin lines indicate 95% CI. SNPs with -log_10_(p) values > 4 deviates from the expected distribution and are associated with CMT. **(B)** Manhattan plot displaying the results from the GWAS based on the Swedish ESS Illumina 170K genotypes. Genome-wide significance is reached for one SNP on chromosome 11 (73,290,522 bp) and nominal association is reached for seven SNPs on chromosomes 11 and 27.

A cohort of Swedish ESS dogs was genotyped for genome-wide association analysis for CMT. A total of 332 individuals (188 cases, 144 controls) and 130,238 SNPs remained in the analysis after quality control filtering. The English Springer Spaniels display an inbreeding coefficient of 0.03±0.05. The ESS cohort showed substantial stratification, mainly due to an outlier group visible in the MDS plot (S1 Fig). The outlier group could potentially be due to genetic mix-in from other breeds. A standard case-control chi-square test resulted in a genomic inflation λ = 2.34, clearly indicating a stratified dataset. The inflation was controlled by removal of an outlier group of 33 individuals, and by mixed model analysis with PCA covariates to correct for residual stratification and cryptic relatedness in the remaining 180 cases and 119 controls (λ = 1.00, Fig 1A). Several loci showed association with CMT, indicating multiple risk factors in the ESS breed (Fig 1B, Table 1). Genome-wide significant association was detected on canine chromosome 11 (SNP BICF2G630310626, chr11:73,290,522, p_raw_ = 5.6x10^-7^, p_perm_ = 0.019). Allele frequencies of this top SNP was also studied investigated in the Swedish outlier group (n = 33), a UK cohort (n = 40) and a Norwegian cohort (n = 15) to investigate a potential overlap in association signal in these minor cohorts. The association with CMT in the original Swedish ESS was however only replicated in the Swedish outlier group, which could indicate an enrichment of this risk variant in the Swedish population (S2 Fig). In addition to the top SNP, seven SNPs located in three genomic regions have p-values deviating from the expected in the QQ-plot (nominal significance threshold at -log p>4.0, Fig 1A). Three SNPs are located on canine chromosome 11, supporting the genome-wide significant locus, and four SNPs are positioned in two loci on chromosome 27. The nominally associated SNPs are listed in Table 1. No SNPs were excluded due to HWE inconsistencies.

**Table 1:** SNPs associated with CMT.

SNP ID	Chr	Position (bp)	Alleles	P	P_perm_	OR (95% CI)	MAF_A_	AF_U_
BICF2G630310626	11	73,290,522	C/T	5.56x10^-7^	0.019	2.76 (1.72–5.57)	0.27	0.12
BICF2G630310945	11	73,599,475	A/G	1.54x10^-6^	0.061	2.18 (1.45–4.26)	0.31	0.17
BICF2G630311035	11	73,684,890	A/G	6.55x10^-6^	0.253	2.01 (1.34–3.83)	0.30	0.18
BICF2G630311065	11	73,716,158	C/T	3.76x10^-6^	0.154	2.05 (1.37–3.95)	0.31	0.18
BICF2P1040993	27	735,281	C/T	2.40x10^-5^	0.505	0.52 (0.25–0.72)	0.40	0.56
BICF2P376878	27	745,156	T/G	1.97x10^-5^	0.682	0.42 (0.18–0.58)	0.38	0.60
BICF2P815910	27	7,683,337	A/G	8.32x10^-6^	0.250	3.01 (1.87–6.49)	0.26	0.11
BICF2P365456	27	7,706,463	A/G	8.30x10^-6^	0.235	2.97 (1.85–6.39)	0.27	0.11

Pperm = Empirical p value 10,000 permutations; OR = Odds Ratio; CI = Confidence interval; MAF_A_ = Minor allele frequency affected; AF_U_ = Allele frequency unaffected

Linkage disequilibrium clumping was used to define the associated regions for further analysis, using both association and LD values to restrict the regions, Table 2. Several of the identified regions overlap with variants associated with different forms of cancer in humans [27]. However, none of the regions contain known genes or GWAS sites for human breast cancer [27].

**Table 2:** Top ten GWAS regions defined by linkage disequilibrium (cut-off p<0.1 and r^2^>0.2).

Chr	Start (bp)	Stop (bp)	Size (Mb)	P	No of genes*
11	72,208,712	74,370,769	2.16	5.56x10^-7^	16
27	4,385,757	10,297,035	5.92	8.30x10^-6^	97
27	250,648	1,143,793	0.89	1.97x10^-5^	24
4	16,015,007	22,457,873	6.45	1.12x10^-4^	55
12	32,400,312	39,306,922	6.91	1.21x10^-4^	43
27	1,411,816	9,487,056	8.09	1.57x10^-4^	181
33	27,745,549	29,722,773	1.98	1.57x10^-4^	35
1	47,466,223	56,984,501	9.53	1.69 x10^-4^	87
6	15,494,678	22,523,760	7.05	1.82x10^-4^	191
X	20,667,479	25,589,819	4.92	2.45x10^-4^	14

*Gene annotations from human genome hg18 (UCSC).

91% of the cases carry at least one risk allele at the three top loci (S3A Fig). The chromosome 11 peak confers a substantial risk (OR = 2.76, 95% CI 1.72–5.57) and accounts for 11.0±7.2% of the phenotypic variance, whereas the three associated regions together explain 28.1±10.0% of the phenotypic variance (S3B Fig). Interestingly, the proportion increases to 34.8±11.0% for the top 5 regions and 38.2±10.8% for the top 10 regions, despite the lack of genome-wide significant association.

### Candidate region re-sequencing

The associated and potentially-associated regions were re-sequenced in 7 ESS dogs selected for optimal variance. The re-sequencing resulted in a coverage of 159x±33x and 90±3% of the target covered by 20x or more. On average 24,500 SNPs and 13,100 short indels were detected in each dog across the 12 Mb sequenced. Nine non-synonymous SNPs were discovered within the top region on chromosome 11, three of these were previously known canine SNPs. No SNPs with predicted deleterious effects were identified within the two nominally associated regions on chromosome 27. The identified SNPs were evaluated for their potential biological function and whether they complied with risk and protective haplotypes in the sequenced dogs. Fifty-four candidate SNPs were selected for genotyping in the ESS cohort. Several larger structural variants were also detected in the top candidate regions, with the majority overlapping repetitive elements or flanking gaps in the genome assembly, indicating alignment difficulties. This was especially evident in the region on chromosome 27:0.7Mb, where two deletions, four duplications and one inversion ranging from 200 bp to 43 kb were detected.

### Genome-wide significant candidate region on chromosome 11

**Fig 2:**
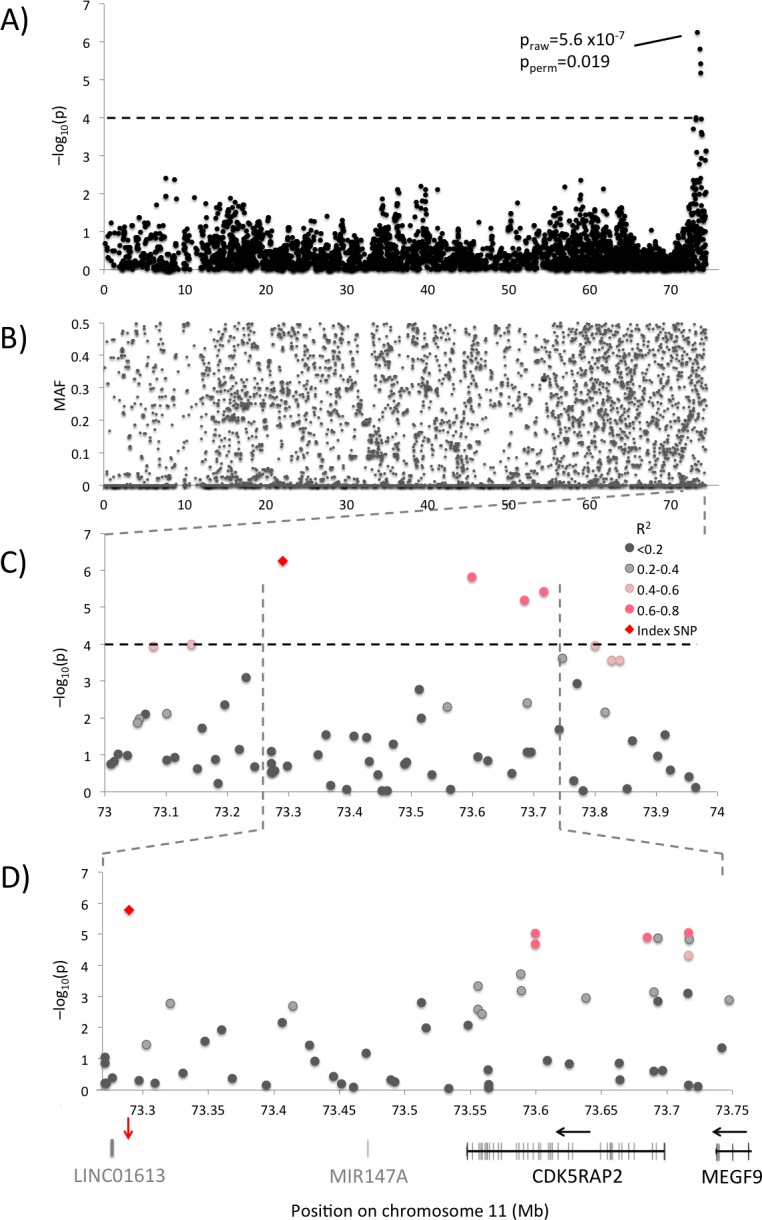
Association results for chromosome 11. **(A)** Association plot and **(B)** Minor allele frequency plot for chromosome 11. **(C)** Candidate region with association results colour-coded according to pair-wise LD (r^2^) with the top SNP (index). **(D)** Candidate region showing association results for the merged and imputed GWAS and sequence candidate SNP dataset with colours according to pair-wise LD (r^2^) with the top SNP. The top haplotype spans a region containing *CDK5RAP2*, *MEGF9* and potentially also *MIR147A* and *LINC01613*. Black arrows indicate direction of transcription and red arrow the top SNP position.

The chromosome 11 candidate locus (Fig 2A and 2C) shows a dispersed minor allele frequency pattern, with no signs of reduced variability due to selective pressure in the region (Fig 2B). Haplotype and LD analysis was performed for the top candidate region (chr11:76.1–76.8Mb, Fig 2D) using a merged dataset including genotypes from the canine SNP chip combined with candidate SNPs identified by sequencing. This dataset was imputed to allow haplotype, LD and association comparisons between markers.

**Fig 3:**
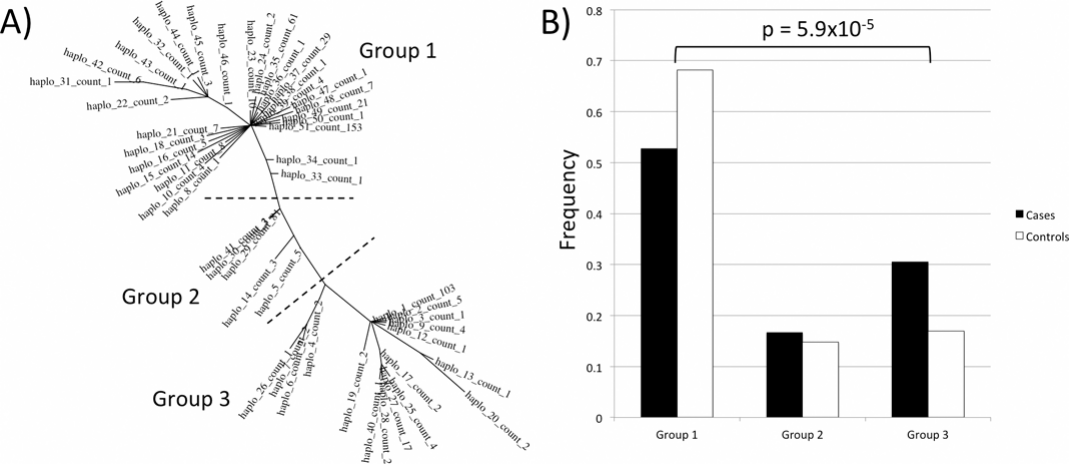
Haplotypes in the chromosome 11 candidate region. **(A)** Phylogenetic tree displaying haplotype relationship of 15 SNPs in the candidate region on chromosome 11. The 51 haplotypes can be formed into three groups based on the tree clusters (separated by dashed lines). **(B)** Case/control frequencies in the three haplotype groups in the ESS cohort. There is a lower proportion of cases in haplotype group 1 compared to group 3.

The 700 kb region on chromosome 11 displayed a complex genetic architecture with a multitude of haplotypes, which persisted when taking potential genotype or imputation errors into account. When investigating only SNPs with signs of association (p<0.001, 15 SNPs), 51 different haplotypes were identified of which 17 were private, indicating an unusually high genotypic diversity in the candidate region. Based on data from Auton *et al* [28], there are four recombination hotspots within the region and the recombination hotspot density is significantly higher in this region compared to the rest of chromosome 11 (p = 0.017), which could be an explanation for the increased diversity in the region. The phylogenetic relationship between the haplotypes was investigated and the haplotypes can be clustered into three groups with 29, 5 and 17 haplotypes in each group (Fig 3A). The haplotype frequencies are 0.59, 0.16 and 0.25 for haplotype group 1, 2 and 3, respectively. Haplotypes belonging to group 3 confer a higher risk for CMT than group 1 (p = 5.9x10^-5^, OR = 2.3, Fig 3B). No significant difference could be established between haplotype group 2 and either group 1 or 3.

The top locus on chromosome 11 could be further defined in the combined GWAS and fine-mapping dataset by markers in high LD (r^2^>0.6) with the top SNP, Fig 2D, restricting the candidate region to approximately 446 kb (73.278–76.723Mb). This region spans the *CDK5RAP2* (CDK5 Regulatory Subunit Associated Protein 2) and parts of the *MEGF9* (Multiple Epidermal Growth Factor-Like Domains Protein 9) gene, both with previous connections to cancer [29,30]. There could potentially also be a microRNA and a lincRNA gene in the region since the human *MIR147A* and *LINC01613* overlap gaps in the dog genome assembly (CanFam 2.0 and CanFam 3.1). Three putative non-synonymous SNPs in *CDK5RAP2* and one SNP in the 3’UTR of *MEGF9* were included in the analysis, but the chr11:73,290,522 top SNP identified in the GWAS remained the most significantly associated after comparisons with potential candidates from re-sequencing, but with a slightly higher p-value after imputation (p = 1.66x10^-6^), Fig 2D). This SNP is located in a small gene desert downstream of *CDK5RAP2*. The region is evolutionary conserved, indicating potential functional importance. The base is evolutionary conserved in 95% of the vertebrates and all mammals evaluated (UCSC genome browser, 100 vertebrates). The SNP is predicted to significantly alter the transcription factor binding abilities for the photoreceptor cell-specific nuclear receptor (PNR/NR2E3, p = 5.3x10^-5^ from TOMTOM [31]), which is specific for the protective allele. PNR/NR2E3 is an orphan nuclear hormone receptor previously reported to have a regulatory role in breast cancer [32,33].

Interestingly, two other SNPs in the candidate region show high association and LD with each other (r^2^ = 0.94) but relatively low LD (r^2^<0.4) with the top SNP, indicating a possibility of two independent genetic risk factors in the area. One of these SNPs produces a non-synonymous change in *CDK5RAP2* (at chr11:73,692,993 bp, grey in Fig 2D). The SNP at chr11:73,692,993 creates a proline to alanine transition, but the amino acid is not well conserved evolutionary and the change is predicted to be benign (score 0.156) when analysed with PolyPhen [34]. It displays strong association to CMT (p = 1.3x10^-5^), but is in moderate LD with the top SNP at chr11:73,290,522 bp (r^2^ = 0.38), and could thus be an alternative genetic risk factor in the region. Analysing the dataset with the chr11:73,290,522 top SNP genotypes as covariates does however remove the association signal in the entire chromosome 11 region (p>0.02), indicating that the associated SNPs are not independent.

### Associated candidate regions on chromosome 27

**Fig 4:**
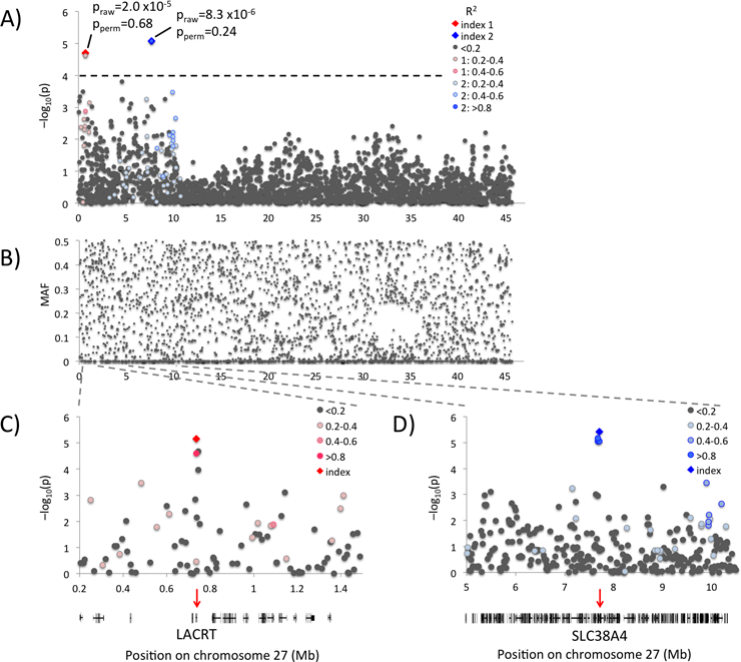
Association results for chromosome 27. **(A)** Chromosome 27 with association results colour-coded according to pair-wise LD (r^2^) with the top two SNPs (index 1 = chr27:745,156 bp and 2 = chr27:7,706,463 bp). **(B)** Minor allele frequency plot over chromosome 27. **(C)** Association results for the merged and imputed GWAS and sequence candidate SNPs dataset at the 0.7 Mb peak. The top SNP (chr27:735,281 bp) is located 418 bp upstream of *LACRT*, SNP position indicated by red arrow. Gene annotations are lifted over from the human genome. **(D)** Association results for the 7.7 Mb region. The top SNP (chr27:7,706,463 bp) is potentially located in an intron of *SLC38A4*.

The entire proximal 13.5 Mb of chromosome 27 shows elevated levels of association, with nominally associated peaks at 0.7 and 7.7 Mb, Fig 4A. An associated region of this size could indicate selection at this site, but the allele frequencies vary and do not suggest decreased genetic variation in the area, Fig 4B. The two loci appear independent with low LD (r^2^ = 0.03) between the top SNPs, Fig 4A. Fine-mapping with additional SNPs did not result in new association signals or restrict the size of either region (Fig 4C and 4D). The 0.7 Mb region contains several large gaps and is poorly annotated in both the CanFam 2.0 and CanFam 3.1 genome assemblies, but the corresponding human region includes 17 genes. Several larger structural variations (SVs) were detected when re-sequencing this region in seven dogs, either reflecting an unstable genomic region or, alternatively, could be indicative of errors in design and sequence alignment due to the incomplete dog genome assembly in this area. After resequencing, genotyping and imputation, the SNP BICF2P1040993 (chr27:735,281 bp) showed the strongest levels of association in the region (p = 6.8x10^-6^). It is located 418 bp upstream of the lacritin gene (*LACRT*, annotated from human hg19), which encodes a glycoprotein involved in tear secretion [35]. *LACRT* expression has also been detected in breast tissue (normal breast tissue, breast cancer tissue and breast cancer cell lines) [36].

After fine-mapping of the 7.7 Mb candidate region, the strongest association is seen for two SNPs located 23 kb apart (BICF2P815910, chr27:7,683,337 and BICF2P365456, chr27:7,706,463). Based on gene annotation in human, this region includes the 5’ part of the amino acid transporter *SLC38A4*, which is known to be imprinted [37]. The SNP with the lowest p-value in this area, BICF2P365456, chr27:7,706,463, is located intronic of the *SLC38A4* gene based on human genome gene annotations (Fig 4D).

### Pathway analysis reveals enrichment of snoRNA and tumour antigen genes

The ten most associated GWAS regions contain excellent candidate genes previously connected to cancer, several of which are novel in breast cancer. We used a PubMed text-based pathway analysis tool (GRAIL) to evaluate gene relationships linking the top ten GWAS loci [38]. Highly significant connections were found for six of the ten regions (p_GRAIL_≤4.6x10^-6^, S1 Table), which all contain small nucleolar RNA (snoRNA) genes. The snoRNAs are involved in post-transcriptional modification of mainly ribosomal RNA and small nuclear RNA. Emerging evidence connect several snoRNAs to cancer [39].

In addition to the associated regions, 117 regions with reduced genetic variability (RGVs) were identified in the ESS cohort (MAF<0.01 over >250 kb), S2 Table. The RGVs cover 2.1% of the genome, and 47 of the RGVs (representing 19.5% of the X chromosome and 1.0% of the genome) are located on the X chromosome. When allowing for more variation (MAF<0.05), RGV regions cover 2.2% of the autosomes and 25.4% of the X chromosome. This is consistent with a lower recombination rate leading to reduced genetic variation on the X chromosome. There is also a bias towards the X chromosome due to a lower marker density (average distance 22.1 kb compared to 13.0 kb for the autosomes). The syntenic human regions were extracted for the 117 regions, of which 99 contain genes and could be evaluated for pathway enrichments. Using GRAIL, 29 RGV regions were significantly connected (p_GRAIL_<0.05), mainly through genes with connections to cancer (14 of the 29 regions). Of these, ten can be classified as tumour antigen genes, including *PAGE2B* (prostate-associated P antigen family, member 2B, p_GRAIL_ = 3.3x10^-9^), *SAGE1* (sarcoma antigen 1, p_GRAIL_ = 5.8x10^-6^), *XAGE5* (X Antigen Family, Member 5, p_GRAIL_ = 8.2x10^-4^), *DUSP21* (Dual Specificity Phosphatase 21, cancer/testis antigen, p_GRAIL_ = 4.5x10^-3^), *CT55* (*CXorf48*, cancer/testis antigen 55, p_GRAIL_ = 3.8x10^-2^) and five melanoma-associated antigens (*MAGEA11*, *MAGED2*, *MAGED4*, *MAGED9* and *MUM1L1*) (S2 Table). The tumour antigen gene products can act as antigens in tumour tissue due to somatic mutations or aberrant expression, which can lead to an immune response. Any altered protein could act as a tumour associated antigen, but according to the T cell-defined tumour antigen peptide database [40] there is an overrepresentation of antigen genes on the X chromosome (22.9% of the unique gene entries 2013 are on X, average distance 4.7 Mb compared to 26.9 Mb in the remaining genome), which could potentially cause a bias towards enrichment in the RGV regions.

Interestingly, when combining the GWAS top ten associated regions together with the RGVs, 41 of the 109 regions (excluding regions without genes) are connected (p_GRAIL_<0.05, S3 Table), including both snoRNAs and tumour associated antigens in both datasets.

## Discussion

In this study we have performed a genome-wide association analysis for canine mammary tumours in the English Springer Spaniel breed. We identified a genome-wide significant peak on chromosome 11 and the candidate region includes a regulator of cyclin-dependent kinase 5 (*CDK5RAP2*). A different cyclin-dependent kinase, *CDKN2A* (p16), has recently been identified in two independent dog cancer GWAS studies, suggesting an important role for these proteins in cancer development [15,16]. The region displays a high genetic diversity, which is evident from the lack of a selective sweep signature, a varied high minor allele frequency and a complex haplotype structure with a high number of haplotypes across the region. The reason for the diversity remains unclear, but there are twice as many recombination hotspots defined within the region than expected based on chromosome 11 average (p = 0.017) [28]. The locus is close to the distal end of chromosome 11 and recombination is known to occur at higher rates close to telomeres, possibly explaining the high haplotype diversity in our candidate region [28]. After resequencing and addition of new candidate SNPs to the study, the associated region could be further refined, encompassing the *CDK5RAP2* gene and parts of *MEGF9*. However, the SNP at chr11:73,290,522bp remains the most associated SNP, and is located within a small gene desert upstream of the *CDK5RAP2* gene. The risk allele disrupts a transcription factor binding motif for PNR/NR2E3, which is an orphan nuclear hormone receptor. It is a regulator of the estrogen receptor 1 (*ESR1*) in ER positive breast cancer cells and interleukin 13Ra2 in ER negative breast cancer cells, regulating tumour growth, cell migration and metastasis [32,33]. High expression levels of PNR/NR2E3 have also been associated with longer recurrence-free survival in breast cancer patients and enhanced response to tamoxifen treatment [32]. The function of the PNR/NR2E3 site in this region is unclear, but the high level of conservation and the presence of a transcription factor binding motif indicate that the associated SNP could have a regulatory function. Alternatively this SNP is in high LD with a causative variant that still remains to be detected. Either of these scenarios suggests that the causative variant is of regulatory nature since the three non-synonymous SNPs in *CDK5RAP2* are not the most or independently associated CMT variants. Non-coding predisposing variants would be in line with the majority of the canine cancer associated germ-line variants discovered so far, which are involved in gene regulation and not directly altering the coding sequence [15,16,41]. Dysregulation of cancer genes is also believed to play a major role in predisposition to human cancer, with the majority of GWAS loci being intronic or intragenic [42], and the variants discovered in this study are therefore likely to be of comparative value. However, pinpointing the exact regulatory variants remains a challenge. If the genetic risk factor in the chromosome 11 region is of regulatory nature, it may act in cis or trans. One method for evaluating a potential trans as well as cis acting regulation would be to correlate transcriptome data with haplotype status for this region. Several strong eQTLs are known for *CDK5RAP2* in humans [43], but the corresponding bases do not seem to be variable in this ESS cohort. It is however possible that different sites may confer a similar mechanism in the dog. The associated SNPs discovered in this study could have eQTL functions and control regulation of *CDK5RAP2*. This would be in concordance with previous studies demonstrating the importance of *CDK5RAP2* expression for maintaining the normal cell cycle checkpoint [30]. The CDK5RAP2 protein has a fundamental role in the centrosome when attaching the mitotic spindle pole to the centrosome during mitosis and is also required for the mitotic spindle checkpoint in response to DNA damage [44]. Mutations in the *CDK5RAP2* gene can cause primary microcephaly due to abnormalities in cell division during neurogenesis [45]. Furthermore, there are indications that *CDK5RAP2* levels can influence cancer treatment response since *CDK5RAP2* knockdown breast cancer cell lines display increased resistance towards paclitaxel and doxorubicin in a study by Zhang *et al* [30]. The germ-line risk factor identified in this study could potentially be utilised to predict treatment outcome when using these chemotherapy drugs, but further studies are needed to establish *CDK5RAP2*’s putative involvement in ESS CMT and the possible influence it may have on treatment regimes. The candidate region also contains parts of the *MEGF9* gene and although no eQTL are reported in the region identified in this study, the GTEx portal shows nearby variants regulating *MEGF9* expression in breast tissue [46].

We have identified three significant CMT associated regions in this study, which account for 28% of the disease risk in the breed. In contrast, the top ten associated loci are predicted to explain 38% of the risk, which indicates that several inherited risk factors play important roles in the development of the disease. In addition to the GWAS loci, regions with a very low variability and therefore undetected by GWAS could also add to the genetic predisposition of CMT. Insurance records show that the Swedish ESS population has a very high CMT risk with 32% of the bitches being affected by ten years of age [20], and it is likely that most individuals in the breed are homozygous for genomic regions containing risk genes increasing the overall CMT risk in the ESS. These regions could have arisen due to random segregation or due to selection of a desired trait. A gene influencing cancer development could therefore be directly involved in the selection or be hitchhiking with the gene selected for. We identified fixed regions covering up to 2.1% of the autosomes and 25.4% of the X chromosome. Although it is expected that genes on the X chromosome might be associated to CMT, the bias towards long fixed regions on the X chromosome could potentially also create spurious findings. The number of regions with reduced variability is smaller than in for instance Rottweilers, Irish wolfhound and greyhounds [15], which indicates a relatively high diversity in the ESS compared to these breeds. This is also reflected in the relatively low inbreeding coefficient (0.03±0.05), which is similar to what has previously been reported in the US ESS population [47] and is probably due to a large population size compared to many breeds.

When analysing possible pathway connections between the genomic regions identified in the GWAS, five snoRNA gene clusters are present in the top regions, with highly significant p-values (p<5x10^-6^ for all linked snoRNA regions). Altered expression levels of snoRNAs have been associated with several cancer forms, including breast cancer and canine mammary tumours [48–50], and elevated snoRNA biogenesis has been found essential for breast cancer tumourigenicity by affecting the tumour suppressor p53’s function [51]. It has been reported that snoRNAs can act as putative oncogenes [48–50,52,53] and regulate other cancer genes. There are also indications that snoRNAs could influence treatment response in breast cancer since altered snoRNA expression levels have been described in Tamoxifen resistant breast cancer cell lines [54].

The regions with reduced genetic variability are strongly enriched for cancer related genes. In particular, tumour antigen genes, such *PAGE2B* (prostate-associated P antigen family, member 2B), sarcoma antigen 1 (*SAGE1*) and several melanoma-associated antigens are overrepresented in the RGV regions. The tumour antigen gene products can act as antigens due to somatic mutations, overexpression or by expression in a cell type where they are normally not expressed which can lead to an activation of the host immune defence. The tumour antigens can also be utilised as biomarkers or in cancer treatment, which is reflected in the large number of ongoing clinical trials focusing on tumour antigens (>1600 in www.clinicaltrials.gov as of Jan 2015). Even though most proteins have the ability to become tumour antigens when mutated, there is an overrepresentation of antigen genes on the X chromosome, which could potentially cause a bias in the pathway analysis reflecting the RGV detection. However, there are also tumour antigen genes in two of the top ten GWAS loci (*MAGEB10* and *DDX43*). There is a substantial overlap of similar genes and pathways in the RGV and GWAS datasets, where tumour antigen genes and snoRNAs link the two datasets and are present in both RGV and GWAS loci. This overlap indicates that the associated and fixed regions affect similar pathways, which are likely influential in CMT development. These results support the theory that both associated and RGV regions contribute to the high rate of CMT in the ESS breed. The fixed regions would increase the base level risk in the breed overall, with genes in the GWAS loci elevating the susceptibility further for individuals carrying the risk genotypes.

This study demonstrates the power of utilising a closed population such as a dog breed for finding genetic susceptibility loci. We have identified a genome-wide significant locus with a promising candidate gene using only about 300 dogs. We note however that several other loci are significantly connected to the identified pathways and also add to the CMT risk explained in the breed. We hypothesise therefore that adding more dogs and markers to the study would bring additional loci to genome-wide significance. It remains however unclear whether our results are applicable to other dog populations. We have investigated other European ESS populations (Norwegian and British), and could see no correlation of the top SNP identified in this study with CMT, but the sample sizes are too small to make any definite conclusions. There is however a trend towards replication in the Swedish outlier group, which would suggest that the risk factor is enriched in Sweden. The accumulation of the risk allele in Sweden is possibly due to an excess of carriers in the population founding the breed in Sweden. It would be of great interest to explore ESS populations from different continents as well as different breeds to investigate if the same genes and pathways are implicated. Furthermore, comparative studies to human breast cancer would be highly prioritised since further studies are required to understand the potential involvement of *CDK5RAP2* and *MEGF9* in breast cancer. Other animal models with mammary tumours could also be considered, such as rats. Similar to dog breeds, rat strains differ in their susceptibility to mammary tumours [55,56]. The associated region on chromosome 11 reported here overlap with rat mammary tumour susceptibility loci [57,58]. Five QTLs in rat, however large, overlap with the associated region [59]; three linked to exposure to tobacco metabolites [60,61] and two linked to estrogen levels [58,62]. Several of the detected GWAS loci overlap with variants associated with different forms of cancer in humans, such as colorectal and prostate cancer, but none of the regions have been reported as significantly associated with breast cancer [27]. We have previously shown that the known human breast cancer genes *BRCA1*, *BRCA2* and *ESR1* are associated with CMT in ESS dogs overlapping with this cohort [25,26], although not as strongly as the risk factors identified in this GWAS study. The similarities in epidemiology, clinical features and genetic predisposition suggest that CMT could be used as a model for breast cancer on many levels. The *CDK5RAP2*, *MEGF9*, snoRNA and tumour antigen pathways identified in this study could thus potentially also play roles in human breast cancer. If so, these could possibly influence treatment response to chemotherapeutic agents and therefore be used to guide the choice of treatment. Further validations, initially in the dog, are certainly necessary to assess whether this hypothesis is true.

### Conclusions

We have performed the first genome-wide study to identify the underlying cause of CMT, which is a spontaneously occurring tumour with many similarities to human breast cancer. We have identified significant CMT association in a region overlapping the *CDK5RAP2* gene. This study further demonstrated the value of CMT as a comparative model for breast cancer for future genetic and clinical studies.

## Materials and Methods

### Ethics statement

All blood and buccal swab samples were collected from English springer spaniel pet dogs with owner’s consent according to the ethically approved protocols of the participating institutions.

### Samples

All blood and buccal swab samples were collected from English springer spaniel pet dogs with owner’s consent according to the ethically approved protocols of the participating institutions. A total of 216 CMT cases and 175 controls were collected. Of these, 336 ESS samples were collected in Sweden (190 cases and 146 controls), 40 in the United Kingdom (18 cases and 22 controls) and 15 in Norway (8 cases and 7 controls).

Swedish ESS blood samples were collected by veterinarians in different veterinary animal hospitals and veterinary clinics throughout Sweden between the years 2005 and 2010 and information was collected regarding possible risk factors for the development of mammary tumours for most dogs (signalment, age of onset, sex, spaying, lactation, use of contraceptives, diet, pregnancy, disease status, and family cancer history) as well as pathology reports and/or other clinical diagnostic information. The average age of diagnosis was 10.8 years, ranging from 5 to 17 years of age. The age of diagnosis is based on the age at time of surgery, which often occurs several years after initial detection of lumps in the mammary glands. 23% of the cases were spayed. Control dogs were over 8 years old and with a confirmed absence of CMT based on palpation of the mammary gland performed by a veterinarian. They were also unaffected by any other form of cancer. 25% of the controls were spayed, with an average age of 6.0 years at time of spaying. When samples were available from siblings only one dog was included to reduce the degree of relatedness in the study cohort. Genomic DNA was extracted from whole blood or buccal swabs using the QIAamp DNA Blood Midi Kit (Qiagen, Hilden, Germany), QIAamp DNA Mini Kit (Qiagen), or salt extraction [63]. 196 of the samples were subsequently whole-genome amplified (GenomePlex Whole Genome Amplification (WGA) Kit, Sigma) due to low DNA amounts. Associated risk allele status for *BRCA1*, *BRCA2* and *ESR1* was available for 278, 281 and 178 of the Swedish ESS dogs, respectively [25,26]. The proportion of dogs carrying at least one risk allele was 98.2% (*BRCA1*), 85.8% (*BRCA2*) and 94.4% (*ESR1*).

### Genome build and annotation

All designs and data analyses were made using the CanFam 2.0 genome build, and the results lifted over to CanFam 3.1. All positions are in CanFam 3.1 unless otherwise specified. Gene annotations were extracted from ENSEMBL [64] and by lift-over from the human genome hg18 and hg19 using UCSC genome lift-over tool [65].

### Genome-wide association mapping

The Illumina 170K canine HD SNP array was used for the genotyping of approximately 174,000 SNPs with a mean genomic interval of 13 kb [66]. The Swedish cohort of 332 samples was used for GWA analysis. Data quality control was performed using the software package PLINK [67], removing SNPs and individuals with a call rate below 90% and SNPs with a minor allele frequency below 1%. A total of 96 SNPs were removed due to platform genotyping inconsistencies. Population stratification was estimated and visualised in multidimensional scaling plots (MDS) using PLINK (S1 Fig) to detect outliers and subgroups in the dataset after removing SNPs in high linkage disequilibrium (LD) (r^2^>0.95). The GCTA software was used to estimate the inbreeding coefficient [68].

Regions associated with CMT were detected by case-control genome-wide association analysis. The EMMAX software [69] was used to calculate association p-values corrected for stratification and cryptic relatedness using mixed model statistics. The two primary eigenvectors calculated by the GCTA software [68] were used as covariates in the analysis to adjust for stratification. The LD-pruned SNP set was used for the estimations of MDS, eigenvectors in GCTA and relationship matrix in EMMAX, whereas the full QC filtered SNP set was used for association testing. Quantile-quantile (QQ)-plots were created in R [70] to assess possible genomic inflation and to establish suggestive significance levels. Permutation testing was performed in GenABEL [71] using mixed model statistics, two eigenvector covariates calculated by GCTA and 10,000 permutations to establish empirical genome-wide corrected p values. Genome-wide significance is considered for p_perm_ ≤ 0.05. Minor allele frequencies were calculated for each cohort (cases and controls) using PLINK [67]. Odds ratios (ORs) and 95% confidence intervals were also calculated from allele frequencies in PLINK [67]. The allele frequencies in domestic animal populations do not always comply with Hardy-Weinberg equilibrium (HWE) due to the non-random mating, but the top SNPs were tested for HWE to exclude SNPs in extreme HW disequilibrium (p≤0.0001), in order to detect possible genotyping errors.

A restricted maximum likelihood (REML) analysis implemented in the GCTA software [68] was used to estimate how much of the phenotypic variance the associated SNPs account for. The two primary eigenvectors were used as covariates, and the prevalence set to 0.36 [20]. The top ten GWAS regions were defined using LD-based clumping in PLINK [67], where ± 5 Mb of the top SNP positions were searched for associated SNPs (p<0.1) in LD (r^2^>0.2) with the top SNP in each region. The regions analysed are listed in Table 1.

### Regions with low genetic variation

The ESS cohort exhibit regions with reduced genetic variability (RGVs), which are fixed or close to fixed for certain alleles in the breed. These regions could contain risk variants contributing to the high incidence of CMT and are undetected in association studies. Regions with MAF<0.01 for >250 kb in the entire cohort without outliers (180 cases and 119 controls) were selected for further analysis. A less strict cut-off, MAF<0.05 for >250 kb, was also used to enable comparisons to other studies.

### Pathway analysis

The top ten GWAS and all RGV regions were evaluated separately and together for pathway enrichment using the GRAIL software [38], which use published scientific abstracts to evaluate connectivity between genomic regions. 50 kb were added to the flanks of the GWAS and RGV regions and coordinates were translated to human genome 18 using UCSC liftover [65]. Gene size correction and PubMed Text (Aug2012) were applied in GRAIL.

### Candidate region re-sequencing

We performed mutational screening of the 14 most associated regions in order to identify disease-causing variants. A homozygous region on chromosome 30 was also included in the sequencing. The regions were targeted by either hybrid selection (NimbleGen Sequence Capture arrays, Roche NimbleGen) followed by re-sequencing using next generation sequencing (Illumina Genome Analyzer II, Illumina), or by PCR of exons and conserved elements and Sanger sequencing. A total of 12 Mb was targeted and sequence capture was performed using an in-house modified protocol [72]. DNA samples from seven ESS dogs (3 cases, 4 controls), selected to carry haplotypes that captured as much genetic variation as possible, were sequenced. Targeted next generation sequencing was used to evaluate the top regions chr11:73.1–73.8Mb and chr27:0.48–0.76Mb whereas Sanger sequencing of selected regions was applied to the chr27:7.6–7.72Mb region. Several software packages were used for sequence data analysis to identify SNPs, indels and copy number variants in the sequenced regions. The BWA package [73] was used for read alignment to the dog reference genome [74], the GATK pipeline for local realignment and quality score recalibration [75], Picard for removal of read clones and to extract statistics (http://picard.sourceforge.net), Samtools for SNP and small indel variant calling and filtering [76], SnpEff to annotate variants [77], the DELLY software suite for detection of structural variants [78], IGV for visualisation of sequences [79] and SeqScoring [80] for evaluation of conservation using data from the 29 mammals project [81]. CodonCode Aligner (CodonCode) was used to evaluate Sanger sequences.

### Candidate SNP genotyping

Additional genotyping was performed for 61 SNPs using iPLEX Gold Mass ARRAY (Sequenom). Fifty-one of the SNPs were selected from the sequencing data as candidate variants for CMT and eleven were top SNPs from the GWAS included for genotype confirmation. Pyrosequencing (Qiagen, Hilden, Germany) was also used for genotyping of two additional candidate SNPs in the *CDK5RAP2* gene [82] and one SNP was genotyped using PCR amplification followed by restriction enzyme cleavage and gel electrophoresis. The SNPs included in the candidate SNP genotyping are listed in S4 Table.

### Imputation

The Illumina 170K canine HD SNP array dataset was merged with the iPLEX Gold Mass ARRAY and Pyrosequencing SNP data using PLINK [67]. Imputation of missing genotypes was performed with the BEAGLE software [83] before evaluating LD and haplotype structure in the candidate regions. Imputed SNP calls with <90% probability were filtered out, and quality control filtering and association analysis was performed on the imputed dataset using PLINK and EMMAX as described previously in the genome-wide association mapping section. An additional GWAS analysis was also performed with the top SNP (Chr11:73,290,522) genotypes as covariates to investigate this SNP’s impact on the remaining loci.

### Haplotype analysis

Pair-wise r^2^–based LD between markers was used to evaluate the size of candidate regions and whether the associated loci were independent. The r^2^ calculations were performed using the Haploview [84] and PLINK software packages [67] on the expanded and imputed dataset. The candidate locus on chromosome 11 was restricted by SNPs with a pairwise r^2^ ≥ 0.6 with the top SNPs. Haplotype analysis was performed using PHASE v2.1.1 [85] to identify haplotypes in the candidate regions. The SeaView software package was used to construct maximum-parsimony phylogenetic trees with bootstrap resampling (1000 permutations) [86]. The designation of clusters was based on branch length. Chi-square statistics were used to evaluate differences between haplotype groups. The associated SNPs within the detected 446 kb region were evaluated with the transcription factor binding motif tool TOMTOM [31] (JASPAR and UniProbe motif databases, p<0.001). The PolyPhen-2 software was used to evaluate the effect of non-synonymous SNPs [34]. Recombination hotspot data was obtained from Auton *et al* [28]. Student’s t-test was applied to evaluate recombination hotspot density.

## Supporting Information

S1 Fig The Swedish ESS cohort forms two clusters, with an outlier group to the right. Multidimensional scaling plot, displaying the first two dimensions, C1 and C2, showing the overall genetic similarity between the individuals in the study. The circled individuals form an outlier group and were removed from further analysis.
(TIF)

S2 Fig Genotype frequencies for the top GWAS SNP (BICF2G630310626, chr11:73,290,522 bp). Genotype frequencies for the risk (C) and protective allele (T) are displayed for the Swedish main (Sw), Swedish outlier (Sw OL), United Kingdom (UK) and Norwegian (Nw) ESS cohorts.
(TIF)

S3 Fig Phenotypic variance explained. **(A)** Genotypes for the top SNPs in the three regions on chromosome 11 and 27 (two regions) displayed for 180 ESS cases. White denotes homozygous for the protective allele, yellow heterozygous and red homozygous for the risk allele. **(B)** Proportion of the phenotypic variance explained by the SNPs in the top regions identified in the genome-wide association analysis. Estimates are displayed for the genome-wide significant region on chromosome 11, the three associated regions on chromosome 11 and 27 (two loci), the top 5 and top 10 regions, respectively. Error bars indicate standard errors.
(TIF)

S1 Table Pathway analysis result for the top ten GWAS candidate regions. Significant p-values (p_GRAIL_<0.05) are indicated in bold.
(DOCX)

S2 Table Regions with reduced genetic variability, including GRAIL pathway analysis results. Significant p-values (p_GRAIL_<0.05) and tumour associated antigen genes are indicated in bold.
(DOCX)

S3 Table GRAIL pathway analysis results from associated regions and regions with reduced genetic variability. Significant p-values (p_GRAIL_<0.05), snoRNA and tumour antigen genes are indicated in bold.
(DOCX)

S4 Table SNPs included in the candidate SNP genotyping. (DOCX)

## References

[1] Torre LA, Siegel RL, Ward EM, Jemal A (2016) Global Cancer Incidence and Mortality Rates and Trends-An Update. Cancer epidemiology, biomarkers & prevention: a publication of the American Association for Cancer Research, cosponsored by the American Society of Preventive Oncology 25: 16–27. 10.1158/1055-9965.EPI-15-0578 26667886

[2] Stevens KN, Vachon CM, Couch FJ (2013) Genetic susceptibility to triple-negative breast cancer. Cancer research 73: 2025–2030. 10.1158/0008-5472.CAN-12-1699 23536562 PMC3654815

[3] Pharoah PD, Dunning AM, Ponder BA, Easton DF (2004) Association studies for finding cancer-susceptibility genetic variants. Nat Rev Cancer 4: 850–860. 15516958 10.1038/nrc1476

[4] Claus EB, Schildkraut JM, Thompson WD, Risch NJ (1996) The genetic attributable risk of breast and ovarian cancer. Cancer 77: 2318–2324. 8635102 10.1002/(SICI)1097-0142(19960601)77:11<2318::AID-CNCR21>3.0.CO;2-Z

[5] Newman B, Austin MA, Lee M, King MC (1988) Inheritance of human breast cancer: evidence for autosomal dominant transmission in high-risk families. Proc Natl Acad Sci U S A 85: 3044–3048. 3362861 10.1073/pnas.85.9.3044 PMC280139

[6] Hollestelle A, Wasielewski M, Martens JW, Schutte M (2010) Discovering moderate-risk breast cancer susceptibility genes. Curr Opin Genet Dev 20: 268–276. 10.1016/j.gde.2010.02.009 20346647

[7] Peng S, Lu B, Ruan W, Zhu Y, Sheng H, et al (2011) Genetic polymorphisms and breast cancer risk: evidence from meta-analyses, pooled analyses, and genome-wide association studies. Breast Cancer Res Treat. 10.1007/s10549-011-1459-5 21445572

[8] Zhang B, Beeghly-Fadiel A, Long J, Zheng W (2011) Genetic variants associated with breast-cancer risk: comprehensive research synopsis, meta-analysis, and epidemiological evidence. The lancet oncology 12: 477–488. 10.1016/S1470-2045(11)70076-6 21514219 PMC3114278

[9] Zheng W, Long J, Gao YT, Li C, Zheng Y, et al (2009) Genome-wide association study identifies a new breast cancer susceptibility locus at 6q25.1. Nature genetics 41: 324–328. 10.1038/ng.318 19219042 PMC2754845

[10] Aloraifi F, Boland MR, Green AJ, Geraghty JG (2015) Gene analysis techniques and susceptibility gene discovery in non-BRCA1/BRCA2 familial breast cancer. Surgical oncology 24: 100–109. 10.1016/j.suronc.2015.04.003 25936246

[11] Drogemuller C, Karlsson EK, Hytonen MK, Perloski M, Dolf G, et al (2008) A mutation in hairless dogs implicates FOXI3 in ectodermal development. Science 321: 1462 10.1126/science.1162525 18787161

[12] Karlsson EK, Baranowska I, Wade CM, Salmon Hillbertz NH, Zody MC, et al (2007) Efficient mapping of mendelian traits in dogs through genome-wide association. Nat Genet 39: 1321–1328. 17906626 10.1038/ng.2007.10

[13] Olsson M, Meadows JR, Truve K, Rosengren Pielberg G, Puppo F, et al A Novel Unstable Duplication Upstream of HAS2 Predisposes to a Breed-Defining Skin Phenotype and a Periodic Fever Syndrome in Chinese Shar-Pei Dogs. PLoS Genet 7: e1001332 10.1371/journal.pgen.1001332 21437276 PMC3060080

[14] Salmon Hillbertz NH, Isaksson M, Karlsson EK, Hellmen E, Pielberg GR, et al (2007) Duplication of FGF3, FGF4, FGF19 and ORAOV1 causes hair ridge and predisposition to dermoid sinus in Ridgeback dogs. Nat Genet 39: 1318–1320. 17906623 10.1038/ng.2007.4

[15] Karlsson EK, Sigurdsson S, Ivansson E, Thomas R, Elvers I, et al (2013) Genome-wide analyses implicate 33 loci in heritable dog osteosarcoma, including regulatory variants near CDKN2A/B. Genome biology 14: R132 10.1186/gb-2013-14-12-r132 24330828 PMC4053774

[16] Shearin AL, Hedan B, Cadieu E, Erich SA, Schmidt EV, et al (2012) The MTAP-CDKN2A locus confers susceptibility to a naturally occurring canine cancer. Cancer epidemiology, biomarkers & prevention: a publication of the American Association for Cancer Research, cosponsored by the American Society of Preventive Oncology 21: 1019–1027. 10.1158/1055-9965.EPI-12-0190-T PMC3392365 22623710

[17] Dodman NH, Karlsson EK, Moon-Fanelli A, Galdzicka M, Perloski M, et al A canine chromosome 7 locus confers compulsive disorder susceptibility. Mol Psychiatry 15: 8–10. 10.1038/mp.2009.111 20029408

[18] Meurs KM, Mauceli E, Lahmers S, Acland GM, White SN, et al Genome-wide association identifies a deletion in the 3' untranslated region of striatin in a canine model of arrhythmogenic right ventricular cardiomyopathy. Hum Genet 128: 315–324. 10.1007/s00439-010-0855-y 20596727 PMC2962869

[19] Wilbe M, Jokinen P, Hermanrud C, Kennedy LJ, Strandberg E, et al (2009) MHC class II polymorphism is associated with a canine SLE-related disease complex. Immunogenetics 61: 557–564. 10.1007/s00251-009-0387-6 19636550

[20] Egenvall A, Bonnett BN, Ohagen P, Olson P, Hedhammar A, et al (2005) Incidence of and survival after mammary tumors in a population of over 80,000 insured female dogs in Sweden from 1995 to 2002. Prev Vet Med 69: 109–127. 15899300 10.1016/j.prevetmed.2005.01.014

[21] Moe L (2001) Population-based incidence of mammary tumours in some dog breeds. J Reprod Fertil Suppl 57: 439–443. 11787188

[22] Vail DM, MacEwen EG (2000) Spontaneously occurring tumors of companion animals as models for human cancer. Cancer investigation 18: 781–792. 11107448 10.3109/07357900009012210

[23] Antuofermo E, Miller MA, Pirino S, Xie J, Badve S, et al (2007) Spontaneous mammary intraepithelial lesions in dogs—a model of breast cancer. Cancer epidemiology, biomarkers & prevention: a publication of the American Association for Cancer Research, cosponsored by the American Society of Preventive Oncology 16: 2247–2256. 10.1158/1055-9965.EPI-06-0932 17982119

[24] Chrisp CE, Spangler WL (1980) The malignant canine mammary tumor as a model for the study of human breast cancer In: Shifrine M, Wilson FD, editors. The Canine as a Biomedical Research Model: Immunological, Hematological and Oncological Aspects. Oak Ridge: National Technical Information Service/Us Department of Commerce pp. 331–349.

[25] Rivera P, Melin M, Biagi T, Fall T, Haggstrom J, et al (2009) Mammary tumor development in dogs is associated with BRCA1 and BRCA2. Cancer Res 69: 8770–8774. 10.1158/0008-5472.CAN-09-1725 19887619

[26] Borge KS, Melin M, Rivera P, Thoresen SI, Webster MT, et al (2013) The ESR1 gene is associated with risk for canine mammary tumours. BMC veterinary research 9: 69 10.1186/1746-6148-9-69 23574728 PMC3637093

[27] Burdett T, Hall PN, Hastings E, Hindorff LA, Junkins HA, et al. (Accessed March 30 2016) The NHGRI-EBI Catalog of published genome-wide association studies. Available at: www.ebiacuk/gwas.

[28] Auton A, Rui Li Y, Kidd J, Oliveira K, Nadel J, et al (2013) Genetic recombination is targeted towards gene promoter regions in dogs. PLoS genetics 9: e1003984 10.1371/journal.pgen.1003984 24348265 PMC3861134

[29] Cunha IW, Carvalho KC, Martins WK, Marques SM, Muto NH, et al (2010) Identification of genes associated with local aggressiveness and metastatic behavior in soft tissue tumors. Translational oncology 3: 23–32. 20165692 10.1593/tlo.09166 PMC2822450

[30] Zhang X, Liu D, Lv S, Wang H, Zhong X, et al (2009) CDK5RAP2 is required for spindle checkpoint function. Cell cycle 8: 1206–1216. 19282672 10.4161/cc.8.8.8205 PMC3820842

[31] Gupta S, Stamatoyannopoulos JA, Bailey TL, Noble WS (2007) Quantifying similarity between motifs. Genome biology 8: R24 17324271 10.1186/gb-2007-8-2-r24 PMC1852410

[32] Park YY, Kim K, Kim SB, Hennessy BT, Kim SM, et al (2012) Reconstruction of nuclear receptor network reveals that NR2E3 is a novel upstream regulator of ESR1 in breast cancer. EMBO molecular medicine 4: 52–67. 10.1002/emmm.201100187 22174013 PMC3376834

[33] Zhao Z, Wang L, Xu W (2014) IL-13Ralpha2 mediates PNR-induced migration and metastasis in ERalpha-negative breast cancer. Oncogene. 10.1038/onc.2014.53 PMC4321963 24747967

[34] Adzhubei IA, Schmidt S, Peshkin L, Ramensky VE, Gerasimova A, et al (2010) A method and server for predicting damaging missense mutations. Nature methods 7: 248–249. 10.1038/nmeth0410-248 20354512 PMC2855889

[35] Sanghi S, Kumar R, Lumsden A, Dickinson D, Klepeis V, et al (2001) cDNA and genomic cloning of lacritin, a novel secretion enhancing factor from the human lacrimal gland. Journal of molecular biology 310: 127–139. 11419941 10.1006/jmbi.2001.4748

[36] Weigelt B, Bosma AJ, van 't Veer LJ (2003) Expression of a novel lacrimal gland gene lacritin in human breast tissues. Journal of cancer research and clinical oncology 129: 735–736. 14574570 10.1007/s00432-003-0514-y PMC12161919

[37] Smith RJ, Dean W, Konfortova G, Kelsey G (2003) Identification of novel imprinted genes in a genome-wide screen for maternal methylation. Genome research 13: 558–569. 12670997 10.1101/gr.781503 PMC430166

[38] Raychaudhuri S, Plenge RM, Rossin EJ, Ng AC, Purcell SM, et al (2009) Identifying relationships among genomic disease regions: predicting genes at pathogenic SNP associations and rare deletions. PLoS genetics 5: e1000534 10.1371/journal.pgen.1000534 19557189 PMC2694358

[39] Martens-Uzunova ES, Olvedy M, Jenster G (2013) Beyond microRNA—novel RNAs derived from small non-coding RNA and their implication in cancer. Cancer letters 340: 201–211. 10.1016/j.canlet.2012.11.058 23376637

[40] Vigneron N, Stroobant V, Van den Eynde BJ, van der Bruggen P (2013) Database of T cell-defined human tumor antigens: the 2013 update. Cancer immunity 13: 15 23882160 PMC3718731

[41] Karyadi DM, Karlins E, Decker B, vonHoldt BM, Carpintero-Ramirez G, et al (2013) A copy number variant at the KITLG locus likely confers risk for canine squamous cell carcinoma of the digit. PLoS genetics 9: e1003409 10.1371/journal.pgen.1003409 23555311 PMC3610924

[42] Welter D, MacArthur J, Morales J, Burdett T, Hall P, et al (2014) The NHGRI GWAS Catalog, a curated resource of SNP-trait associations. Nucleic acids research 42: D1001–1006. 10.1093/nar/gkt1229 24316577 PMC3965119

[43] Zhang L, Kim S (2014) Learning gene networks under SNP perturbations using eQTL datasets. PLoS computational biology 10: e1003420 10.1371/journal.pcbi.1003420 24586125 PMC3937098

[44] Barr AR, Kilmartin JV, Gergely F (2010) CDK5RAP2 functions in centrosome to spindle pole attachment and DNA damage response. The Journal of cell biology 189: 23–39. 10.1083/jcb.200912163 20368616 PMC2854379

[45] Woods CG, Bond J, Enard W (2005) Autosomal recessive primary microcephaly (MCPH): a review of clinical, molecular, and evolutionary findings. American journal of human genetics 76: 717–728. 15806441 10.1086/429930 PMC1199363

[46] The GTEx Consortium (2013) The Genotype-Tissue Expression (GTEx) project. Nature genetics 45: 580–585. 10.1038/ng.2653 23715323 PMC4010069

[47] Calboli FC, Sampson J, Fretwell N, Balding DJ (2008) Population structure and inbreeding from pedigree analysis of purebred dogs. Genetics 179: 593–601. 10.1534/genetics.107.084954 18493074 PMC2390636

[48] von Deetzen MC, Schmeck BT, Gruber AD, Klopfleisch R (2014) Malignancy Associated MicroRNA Expression Changes in Canine Mammary Cancer of Different Malignancies. ISRN veterinary science 2014: 148597 10.1155/2014/148597 25002976 PMC4060554

[49] Mourtada-Maarabouni M, Pickard MR, Hedge VL, Farzaneh F, Williams GT (2009) GAS5, a non-protein-coding RNA, controls apoptosis and is downregulated in breast cancer. Oncogene 28: 195–208. 10.1038/onc.2008.373 18836484

[50] Gee HE, Buffa FM, Camps C, Ramachandran A, Leek R, et al (2011) The small-nucleolar RNAs commonly used for microRNA normalisation correlate with tumour pathology and prognosis. British journal of cancer 104: 1168–1177. 10.1038/sj.bjc.6606076 21407217 PMC3068486

[51] Su H, Xu T, Ganapathy S, Shadfan M, Long M, et al (2014) Elevated snoRNA biogenesis is essential in breast cancer. Oncogene 33: 1348–1358. 10.1038/onc.2013.89 23542174

[52] Dong XY, Guo P, Boyd J, Sun X, Li Q, et al (2009) Implication of snoRNA U50 in human breast cancer. Journal of genetics and genomics = Yi chuan xue bao 36: 447–454. 10.1016/S1673-8527(08)60134-4 19683667 PMC2854654

[53] Mei YP, Liao JP, Shen J, Yu L, Liu BL, et al (2012) Small nucleolar RNA 42 acts as an oncogene in lung tumorigenesis. Oncogene 31: 2794–2804. 10.1038/onc.2011.449 21986946 PMC4966663

[54] Huber-Keener KJ, Liu X, Wang Z, Wang Y, Freeman W, et al (2012) Differential gene expression in tamoxifen-resistant breast cancer cells revealed by a new analytical model of RNA-Seq data. PloS one 7: e41333 10.1371/journal.pone.0041333 22844461 PMC3402532

[55] Colletti JA 2nd, Leland-Wavrin KM, Kurz SG, Hickman MP, Seiler NL, et al (2014) Validation of six genetic determinants of susceptibility to estrogen-induced mammary cancer in the rat and assessment of their relevance to breast cancer risk in humans. G3 4: 1385–1394. 10.1534/g3.114.011163 24875630 PMC4132170

[56] Isaacs JT (1986) Genetic control of resistance to chemically induced mammary adenocarcinogenesis in the rat. Cancer research 46: 3958–3963. 3089584

[57] Stieber D, Piessevaux G, Riviere M, Laes JF, Quan X, et al (2007) Isolation of two regions on rat chromosomes 5 and 18 affecting mammary cancer susceptibility. International journal of cancer Journal international du cancer 120: 1678–1683. 17230524 10.1002/ijc.22400

[58] Schaffer BS, Lachel CM, Pennington KL, Murrin CR, Strecker TE, et al (2006) Genetic bases of estrogen-induced tumorigenesis in the rat: mapping of loci controlling susceptibility to mammary cancer in a Brown Norway x ACI intercross. Cancer research 66: 7793–7800. 16885383 10.1158/0008-5472.CAN-06-0143

[59] Shimoyama M, De Pons J, Hayman GT, Laulederkind SJ, Liu W, et al (2015) The Rat Genome Database 2015: genomic, phenotypic and environmental variations and disease. Nucleic acids research 43: D743–750. 10.1093/nar/gku1026 25355511 PMC4383884

[60] Samuelson DJ, Hesselson SE, Aperavich BA, Zan Y, Haag JD, et al (2007) Rat Mcs5a is a compound quantitative trait locus with orthologous human loci that associate with breast cancer risk. Proceedings of the National Academy of Sciences of the United States of America 104: 6299–6304. 17404222 10.1073/pnas.0701687104 PMC1847458

[61] Samuelson DJ, Haag JD, Lan H, Monson DM, Shultz MA, et al (2003) Physical evidence of Mcs5, a QTL controlling mammary carcinoma susceptibility, in congenic rats. Carcinogenesis 24: 1455–1460. 12844486 10.1093/carcin/bgg112

[62] Schaffer BS, Leland-Wavrin KM, Kurz SG, Colletti JA, Seiler NL, et al (2013) Mapping of three genetic determinants of susceptibility to estrogen-induced mammary cancer within the Emca8 locus on rat chromosome 5. Cancer prevention research 6: 59–69. 10.1158/1940-6207.CAPR-12-0346-T 23151807 PMC3536887

[63] Miller SA, Dykes DD, Polesky HF (1988) A simple salting out procedure for extracting DNA from human nucleated cells. Nucleic acids research 16: 1215 3344216 10.1093/nar/16.3.1215 PMC334765

[64] www.ensembl.org.

[65] www.genome.ucsc.edu/cgi-bin/hgLiftOver.

[66] Vaysse A, Ratnakumar A, Derrien T, Axelsson E, Rosengren Pielberg G, et al (2011) Identification of genomic regions associated with phenotypic variation between dog breeds using selection mapping. PLoS genetics 7: e1002316 10.1371/journal.pgen.1002316 22022279 PMC3192833

[67] Purcell S, Neale B, Todd-Brown K, Thomas L, Ferreira MA, et al (2007) PLINK: a tool set for whole-genome association and population-based linkage analyses. Am J Hum Genet 81: 559–575. 17701901 10.1086/519795 PMC1950838

[68] Yang J, Lee SH, Goddard ME, Visscher PM (2011) GCTA: a tool for genome-wide complex trait analysis. American journal of human genetics 88: 76–82. 10.1016/j.ajhg.2010.11.011 21167468 PMC3014363

[69] Kang HM, Sul JH, Service SK, Zaitlen NA, Kong SY, et al (2010) Variance component model to account for sample structure in genome-wide association studies. Nature genetics 42: 348–354. 10.1038/ng.548 20208533 PMC3092069

[70] R Development Core Team. (2008) R: A language and environment for statistical computing: R Foundation for Statistical Computing, Vienna, Austria.

[71] Aulchenko YS, Ripke S, Isaacs A, van Duijn CM (2007) GenABEL: an R library for genome-wide association analysis. Bioinformatics 23: 1294–1296. 17384015 10.1093/bioinformatics/btm108

[72] Wade CM, Giulotto E, Sigurdsson S, Zoli M, Gnerre S, et al (2009) Genome sequence, comparative analysis, and population genetics of the domestic horse. Science 326: 865–867. 10.1126/science.1178158 19892987 PMC3785132

[73] Li H, Durbin R (2009) Fast and accurate short read alignment with Burrows-Wheeler transform. Bioinformatics 25: 1754–1760. 10.1093/bioinformatics/btp324 19451168 PMC2705234

[74] Lindblad-Toh K, Wade CM, Mikkelsen TS, Karlsson EK, Jaffe DB, et al (2005) Genome sequence, comparative analysis and haplotype structure of the domestic dog. Nature 438: 803–819. 16341006 10.1038/nature04338

[75] McKenna A, Hanna M, Banks E, Sivachenko A, Cibulskis K, et al The Genome Analysis Toolkit: a MapReduce framework for analyzing next-generation DNA sequencing data. Genome Res 20: 1297–1303. 10.1101/gr.107524.110 20644199 PMC2928508

[76] Li H, Handsaker B, Wysoker A, Fennell T, Ruan J, et al (2009) The Sequence Alignment/Map format and SAMtools. Bioinformatics 25: 2078–2079. 10.1093/bioinformatics/btp352 19505943 PMC2723002

[77] Cingolani P, Platts A, Wang le L, Coon M, Nguyen T, et al (2012) A program for annotating and predicting the effects of single nucleotide polymorphisms, SnpEff: SNPs in the genome of Drosophila melanogaster strain w1118; iso-2; iso-3. Fly 6: 80–92. 10.4161/fly.19695 22728672 PMC3679285

[78] Rausch T, Zichner T, Schlattl A, Stutz AM, Benes V, et al (2012) DELLY: structural variant discovery by integrated paired-end and split-read analysis. Bioinformatics 28: i333–i339. 10.1093/bioinformatics/bts378 22962449 PMC3436805

[79] Robinson JT, Thorvaldsdòttis H, Winckler W, Guttman M, Lander ES, et al (2011) Integrative genomics viewer. Nat Biotechnol 29: 24–26. 10.1038/nbt.1754 21221095 PMC3346182

[80] Truvé K, Eriksson O, Norling M, Wilbe M, Mauceli E, et al (2011) SEQscoring: a tool to facilitate the interpretation of data generated with next generation sequencing technologies. EMBnetjournal 17.1: 38–45.

[81] Lindblad-Toh K, Garber M, Zuk O, Lin MF, Parker BJ, et al (2011) A high-resolution map of human evolutionary constraint using 29 mammals. Nature 478: 476–482. 10.1038/nature10530 21993624 PMC3207357

[82] Royo JL, Hidalgo M, Ruiz A (2007) Pyrosequencing protocol using a universal biotinylated primer for mutation detection and SNP genotyping. Nature protocols 2: 1734–1739. 17641638 10.1038/nprot.2007.244

[83] Browning BL, Browning SR (2009) A unified approach to genotype imputation and haplotype-phase inference for large data sets of trios and unrelated individuals. American journal of human genetics 84: 210–223. 10.1016/j.ajhg.2009.01.005 19200528 PMC2668004

[84] Barrett JC, Fry B, Maller J, Daly MJ (2005) Haploview: analysis and visualization of LD and haplotype maps. Bioinformatics 21: 263–265. 15297300 10.1093/bioinformatics/bth457

[85] Stephens M, Scheet P (2005) Accounting for decay of linkage disequilibrium in haplotype inference and missing-data imputation. American journal of human genetics 76: 449–462. 15700229 10.1086/428594 PMC1196397

[86] Gouy M, Guindon S, Gascuel O (2010) SeaView version 4: A multiplatform graphical user interface for sequence alignment and phylogenetic tree building. Molecular biology and evolution 27: 221–224. 10.1093/molbev/msp259 19854763

