# Supplementary figures and images for: Genome-Wide Analysis Identifies Germ-Line Risk Factors Associated with Canine Mammary Tumours
Source: PLoS Genet. 2016 May 9;12(5):e1006029. doi: 10.1371/journal.pgen.1006029 (PMC4861258; doi:10.1371/journal.pgen.1006029)

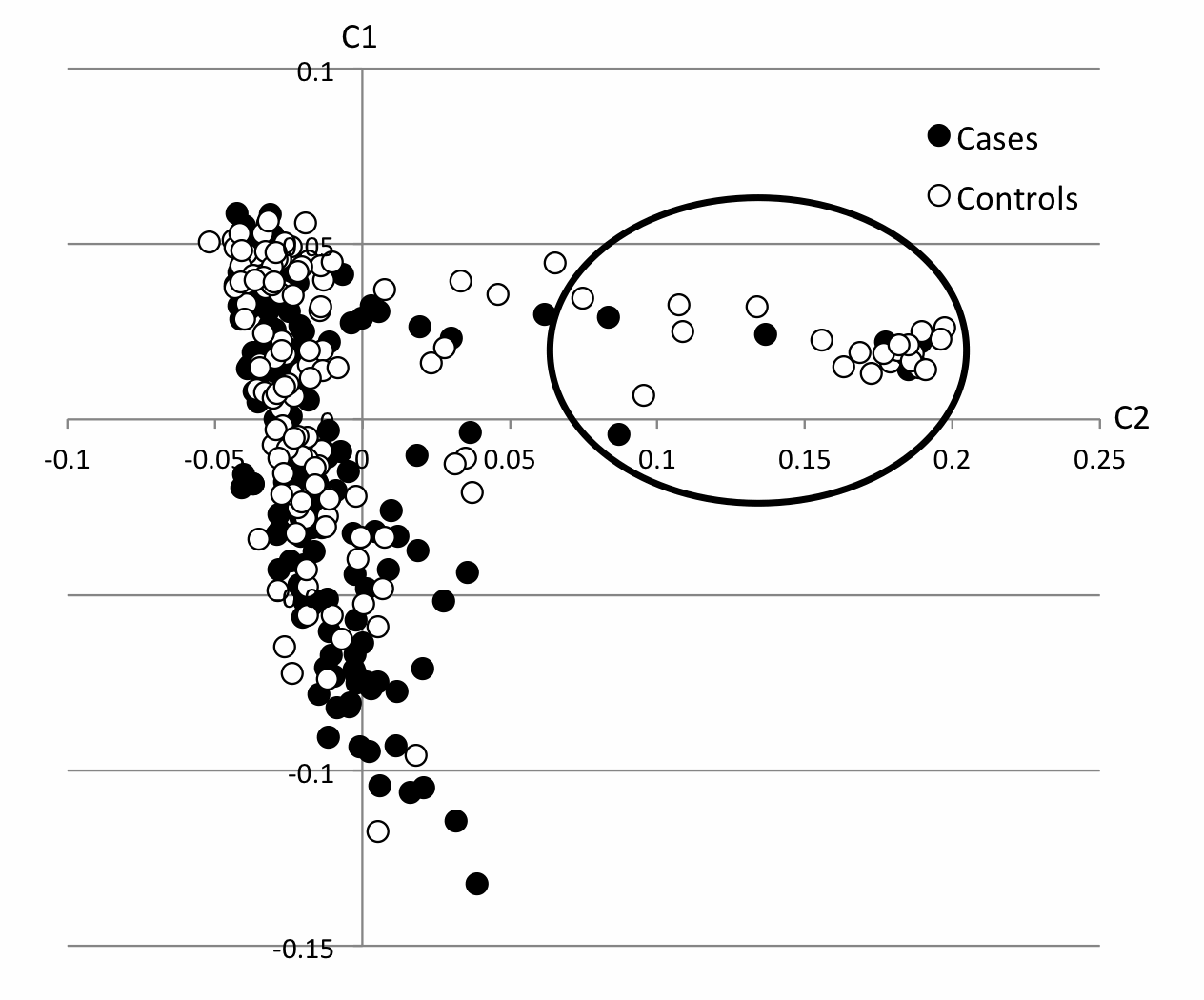

Supplement: S1 Fig — Multidimensional scaling plot, displaying the first two dimensions, C1 and C2, showing the overall genetic similarity between the individuals in the study. The circled individuals form an outlier group and were removed from further analysis. (TIF) [file pgen.1006029.s001.tif]

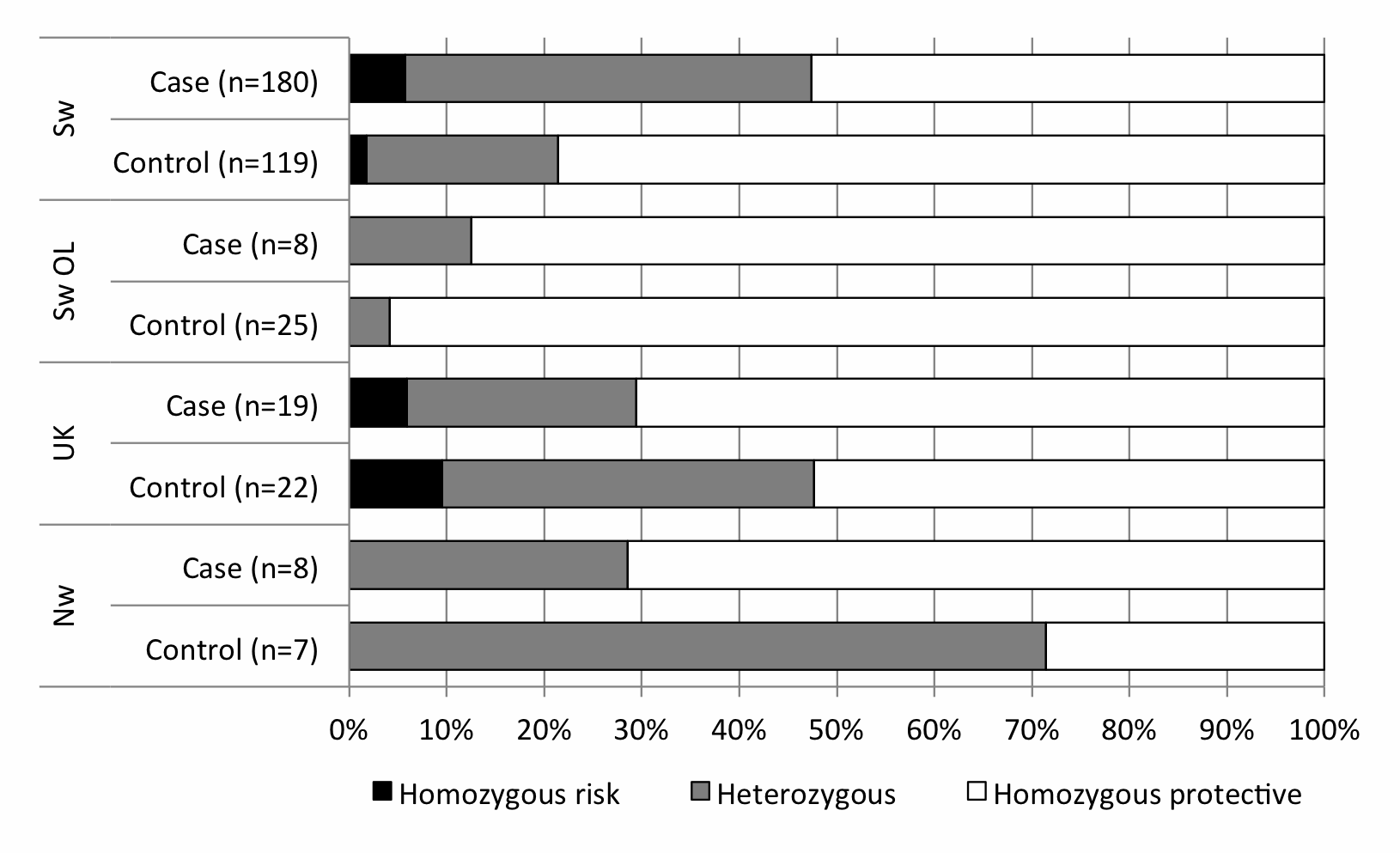

Supplement: S2 Fig — Genotype frequencies for the risk (C) and protective allele (T) are displayed for the Swedish main (Sw), Swedish outlier (Sw OL), United Kingdom (UK) and Norwegian (Nw) ESS cohorts. (TIF) [file pgen.1006029.s002.tif]

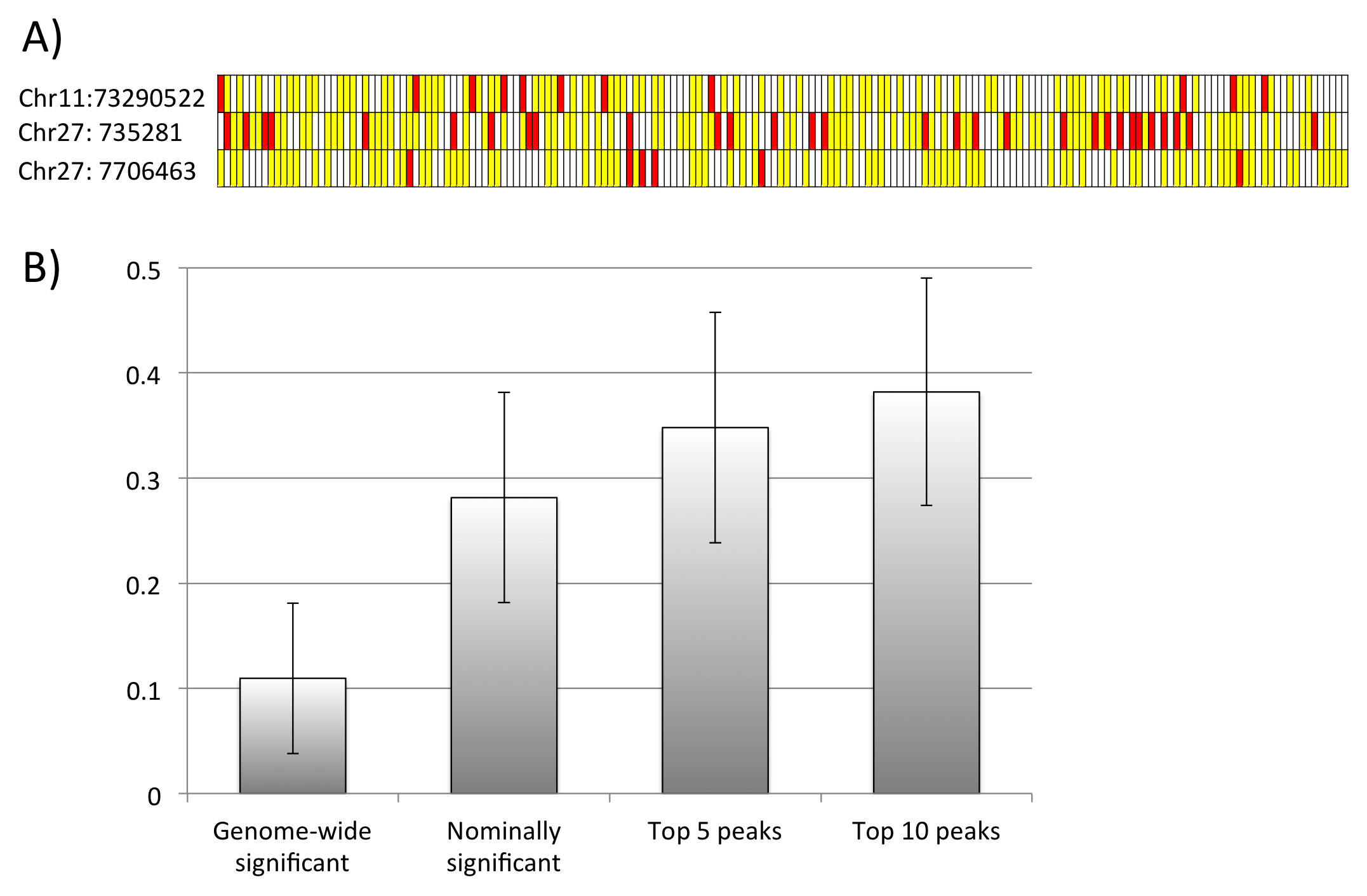

Supplement: S3 Fig — (A) Genotypes for the top SNPs in the three regions on chromosome 11 and 27 (two regions) displayed for 180 ESS cases. White denotes homozygous for the protective allele, yellow heterozygous and red homozygous for the risk allele. (B) Proportion of the phenotypic variance explained by the SNPs in the top regions identified in the genome-wide association analysis. Estimates are displayed for the genome-wide significant region on chromosome 11, the three associated regions on chromosome 11 and 27 (two loci), the top 5 and top 10 regions, respectively. Error bars indicate standard errors. (TIF) [file pgen.1006029.s003.tif]
