# Supplementary material for: Genome-Wide Analysis Identifies Germ-Line Risk Factors Associated with Canine Mammary Tumours
Source: PLoS Genet. 2016 May 9;12(5):e1006029. doi: 10.1371/journal.pgen.1006029 (PMC4861258; doi:10.1371/journal.pgen.1006029)
Supplement: S1 Table — Significant p-values (pGRAIL<0.05) are indicated in bold. (DOCX) [file pgen.1006029.s004.docx]

**Table S1.** Pathway analysis result for the top ten GWAS candidate regions. Significant p-values (p_GRAIL_<0.05) are indicated in bold.

| Region | Chr | Positions (bp) | GRAIL p-value | Candidate gene(s) |
| --- | --- | --- | --- | --- |
| 1 | 11 | 72208712-74370769 | 0.42 | *CDK5RAP2* |
| 2 | 27 | 4385757-10297035 | **2.1x10^-7^** | *SNORA2A, SNORA2B, SNORA34* |
| 3 | 27 | 250648-1143793 | 0.17 | *DCD* |
| 4 | 4 | 16015007-22457873 | **4.6x10^-6^** | *SNORD98* |
| 5 | 12 | 32400312-39306922 | 0.37 | *COX7A2* |
| 6 | 27 | 1411816-9487056 | **4.1x10^-7^** | *SNORA2A, SNORA2B, SNORA34* |
| 7 | 33 | 27745549-29722773 | **8.3x10^-7^** | *SNORA4, SNORA7B, SNORA58* |
| 8 | 1 | 47466223-56984501 | **1.5x10^-7^** | *SNORA20, SNORA29* |
| 9 | 6 | 15494678-22523760 | **1.3x10^-7^** | *SNORA30* |
| 10 | X | 20667479-25589819 | 0.13 | *MAGEB18* |
