# Supplementary material for: Genome-Wide Analysis Identifies Germ-Line Risk Factors Associated with Canine Mammary Tumours
Source: PLoS Genet. 2016 May 9;12(5):e1006029. doi: 10.1371/journal.pgen.1006029 (PMC4861258; doi:10.1371/journal.pgen.1006029)
Supplement: S2 Table — Significant p-values (pGRAIL<0.05) and tumour associated antigen genes are indicated in bold. (DOCX) [file pgen.1006029.s005.docx]

**Table S2.** Regions with reduced genetic variability, including GRAIL pathway analysis results. Significant p-values (p_GRAIL_<0.05) and tumour associated antigen genes are indicated in bold.

| Chr | Position (bp) | Size (kb) | GRAIL p-value | GRAIL candidate gene(s) |
| --- | --- | --- | --- | --- |
| X | 46710296-47429085 | 730 | **3.3x10^-9^** | ***PAGE2B*** |
| X | 75888334-76308456 | 423 | **6.1x10^-9^** | *TCEAL6* |
| X | 118350131-118061542 | 307 | **1.3x10^-6^** | ***MAGEA11*** |
| X | 77248634-77520291 | 271 | **3.9x10^-6^** | *CXorf39* |
| X | 106219851-106525002 | 307 | **5.8x10^-6^** | ***SAGE1*** |
| X | 58753513-59057396 | 306 | **1.6x10^-6^** | *ZDHHC15* |
| X | 30799052-31073968 | 275 | **1.7x10^-5^** | *CXorf59* |
| X | 47937319-49621943 | 4575 | **5.3x10^-5^** | *SPIN3* |
| X | 46454278-46710296 | 305 | **1.1x10^-4^** | ***MAGED2*** |
| X | 119817565-120668042 | 850 | **1.2x10^-4^** | ***MAGEA4*** |
| X | 117531577-117920821 | 344 | **1.2x10^-4^** | ***MAGEA9*** |
| X | 57879733-58373982 | 493 | **1.3x10^-4^** | *KIAA2022* |
| X | 44743230-45351987 | 629 | **8.2x10^-4^** | ***XAGE5*** |
| X | 116185044-116454153 | 269 | **1.5x10^-3^** | *FMR1* |
| 31 | 9567-306904 | 297 | **2.2x10^-3^** | *CGGBP1* |
| X | 79480802-79804494 | 324 | **2.7x10^-3^** | ***MUM1L1*** |
| X | 80502409-80786587 | 285 | **3.5x10^-3^** | *NUP62CL* |
| 18 | 1325939-1685600 | 357 | **4.5x10^-3^** | *ZPBP* |
| X | 38681347-38980925 | 300 | **4.5x10^-3^** | ***DUSP21*** |
| 6 | 23943526-24239505 | 296 | **0.014** | *ZP2* |
| 34 | 14251381-14523727 | 272 | **0.016** | *DNAJC19* |
| 18 | 25079568-25344868 | 264 | **0.019** | *SCGB1C1* |
| 3 | 18397331-18779480 | 382 | **0.021** | *LOC153364* |
| X | 51967352-52719387 | 751 | **0.022** | *OPHN1* |
| 25 | 1023622-1285681 | 262 | **0.022** | *CBR4* |
| 11 | 11447189-11881605 | 437 | **0.034** | *FTMT* |
| X | 38280103-38559598 | 280 | **0.035** | *FUNDC1* |
| X | 105623414-106012466 | 394 | **0.038** | ***CXorf48*** |
| X | 52719387-53254068 | 534 | **0.043** | *OPHN1* |
| X | 119113226-119376597 | 263 | **0.047** | *CD99L2* |
| 20 | 749186-1000681 | 252 | 0.050 | *UROC1* |
| X | 40516553-41108263 | 592 | 0.056 | *ZNF41* |
| X | 53726107-54152257 | 429 | 0.062 | *EDA* |
| 1 | 114675833-114995388 | 320 | 0.065 | *C19orf15* |
| X | 51501813-51938955 | 441 | 0.069 | *AR* |
| X | 49724985-51501813 | 1776 | 0.070 | *ZC3H12B* |
| 15 | 33767163-34118007 | 350 | 0.078 | *MRPL42* |
| 11 | 1307711-1803176 | 498 | 0.087 | *LOC51149* |
| 1 | 102208545-102497770 | 289 | 0.096 | *TMEM190* |
| X | 65713151-66001364 | 288 | 0.12 | *APOOL* |
| 6 | 40408850-40743593 | 334 | 0.12 | *WDR77* |
| 2 | 42981617-43260700 | 279 | 0.15 | *SLC38A9* |
| 10 | 368251-658036 | 292 | 0.15 | *RPL41* |
| 11 | 51577906-51889766 | 311 | 0.16 | *KIAA1539* |
| 23 | 29684535-29961034 | 276 | 0.17 | *ACAD11* |
| 30 | 2049048-2340529 | 291 | 0.17 | *ARHGAP11A* |
| 10 | 15769324-16559368 | 791 | 0.18 | *GLIPR1L1* |
| 6 | 25046567-25315244 | 269 | 0.18 | *GPR139* |
| 7 | 24610867-24887749 | 276 | 0.26 | *RABGAP1L* |
| 30 | 6203662-6457451 | 254 | 0.26 | *RABGAP1L* |
| 13 | 48280354-48558065 | 278 | 0.26 | *KIAA1211* |
| 2 | 4174249-5965377 | 1725 | 0.27 | *ACBD5* |
| 6 | 39754441-40035536 | 281 | 0.27 | *HAGHL* |
| 6 | 24642080-24893954 | 252 | 0.28 | *PDILT* |
| 25 | 1498628-1918540 | 420 | 0.28 | *LHFP* |
| 16 | 6788390-7089736 | 301 | 0.28 | *TRY6* |
| 2 | 57402088-57686136 | 290 | 0.29 | *KIAA0888* |
| 26 | 12829997-13095927 | 266 | 0.29 | *MED13L* |
| X | 64514115-64931709 | 416 | 0.30 | *CYLC1* |
| X | 5824937-6093509 | 269 | 0.30 | *FAM9B* |
| 7 | 55897066-56214015 | 317 | 0.32 | *ASXL3* |
| X | 45427830-46112104 | 684 | 0.34 | *PHF8* |
| 2 | 36185061-36568688 | 384 | 0.35 | *RELL2* |
| X | 115925863-116185044 | 259 | 0.36 | *UBA52* |
| 8 | 73837469-74280399 | 443 | 0.37 | *ADAM6* |
| 8 | 73558627-73837469 | 279 | 0.37 | *ADAM6* |
| 17 | 21439162-21710469 | 395 | 0.39 | *ZNF512* |
| 22 | 16490982-16913009 | 422 | 0.40 | *TDRD3* |
| X | 35075754-35804729 | 729 | 0.46 | *USP9X* |
| 16 | 1495994-1847408 | 352 | 0.46 | *PDIA4* |
| X | 47429085-47810535 | 383 | 0.51 | *KPNA3* |
| 11 | 25593202-25856947 | 267 | 0.51 | *C5orf5* |
| 14 | 198686-561549 | 363 | 0.56 | *HIST3H2BB* |
| 20 | 41422789-41706380 | 284 | 0.56 | *TMEM103* |
| 7 | 39339132-39659508 | 320 | 0.57 | *LBR* |
| X | 14479949-14732776 | 253 | 0.57 | *CDKL5* |
| X | 36863814-37226645 | 363 | 0.57 | *PPP1R2P9* |
| 21 | 269072-521913 | 252 | 0.62 | *PGR* |
| X | 87546053-87856303 | 310 | 0.65 | *IL13RA2* |
| 21 | 20945220-21242465 | 297 | 0.66 | *INTS4* |
| 37 | 11858899-12383891 | 525 | 0.75 | *NBEAL1* |
| 19 | 19783318-20184799 | 400 | 0.77 | *MAD2L1* |
| 10 | 2992717-3374187 | 381 | 0.79 | *SLC16A7* |
| X | 43820164-44186161 | 366 | 0.79 | *BMP15* |
| 3 | 15262164-15565051 | 303 | 0.82 | *C5orf21* |
| X | 67297808-67649939 | 352 | 0.84 | *DACH2* |
| 7 | 28513627-28792399 | 279 | 0.85 | *C1orf156* |
| 14 | 6291022-6612597 | 322 | 0.87 | *COPG2* |
| X | 81690841-81953040 | 262 | 0.89 | *ATG4A* |
| 1 | 111058830-111358874 | 300 | 0.90 | *ZNF155* |
| X | 97169263-97443176 | 278 | 0.94 | *WDR40C* |
| 38 | 3002255-3281511 | 279 | 0.95 | *KCNT2* |
| X | 109240610-109563872 | 323 | 0.96 | *F9* |
| X | 104228144-104515245 | 287 | 0.97 | *GPC3* |
| 25 | 19663132-19916849 | 254 | 0.99 | *C15orf54* |
| 11 | 10504695-10819660 | 308 | 0.99 | *PRR16* |
| 26 | 25026683-25882382 | 895 | 1.0 | *SLC5A4* |
| 1 | 69332109-69663040 | 331 | 1.0 | *AKAP7* |
| 30 | 1235869-1945969 | 709 | 1.0 | *FMN1* |
| 26 | 25987369-26807365 | 818 | N/A | - |
| 11 | 9182677-9844519 | 661 | N/A | - |
| X | 39329154-39916498 | 587 | N/A | - |
| X | 89246507-89666610 | 420 | N/A | - |
| X | 85979587-86376335 | 396 | N/A | - |
| 1 | 79976257-80284525 | 308 | N/A | - |
| 16 | 4725984-5016422 | 290 | N/A | - |
| 18 | 3404120-3688106 | 284 | N/A | - |
| 37 | 4378284-4656997 | 279 | N/A | - |
| 9 | 16733839-17008126 | 275 | N/A | - |
| 17 | 3050615-3321836 | 271 | N/A | - |
| 8 | 2183267-2449883 | 271 | N/A | - |
| 2 | 341839-609856 | 268 | N/A | - |
| 11 | 48536728-48803783 | 266 | N/A | - |
| X | 108091441-108356181 | 265 | N/A | - |
| 20 | 44246949-44507140 | 260 | N/A | - |
| 7 | 24887749-25142403 | 255 | N/A | - |
| X | 93355248-93605403 | 250 | N/A | - |
