# Supplementary material for: Genome-Wide Analysis Identifies Germ-Line Risk Factors Associated with Canine Mammary Tumours
Source: PLoS Genet. 2016 May 9;12(5):e1006029. doi: 10.1371/journal.pgen.1006029 (PMC4861258; doi:10.1371/journal.pgen.1006029)
Supplement: S4 Table — (DOCX) [file pgen.1006029.s007.docx]

**Table S4.** SNPs included in the candidate SNP genotyping.

| Chr | Position (bp) | Alleles |
| --- | --- | --- |
| 4 | 20801409 | G/A |
| 4 | 20838105 | T/G |
| 4 | 20921986 | G/A |
| 4 | 20976740 | G/T |
| 4 | 20977800 | A/G |
| 4 | 20977918 | T/C |
| 4 | 20987402 | G/A |
| 4 | 21046388 | A/G |
| 4 | 21094745 | A/G |
| 4 | 21120848 | G/A |
| 4 | 21229626 | A/G |
| 4 | 21369334 | C/T |
| 4 | 21558553 | T/C |
| 4 | 21605934 | T/C |
| 4 | 21666366 | A/G |
| 4 | 21917298 | C/G |
| 4 | 21964003 | A/G |
| 4 | 22005009 | A/G |
| 4 | 22078555 | A/G |
| 4 | 22422360 | C/A |
| 4 | 22475405 | T/C |
| 4 | 22488624 | T/C |
| 4 | 22563809 | G/A |
| 4 | 22563835 | G/A |
| 4 | 22606984 | T/C |
| 6 | 42010325 | A/G |
| 11 | 73135839 | A/G |
| 11 | 73198863 | A/G |
| 11 | 73239955 | G/A |
| 11 | 73303455 | A/C |
| 11 | 73324859 | A/G |
| 11 | 73555768 | G/A |
| 11 | 73555948 | A/G |
| 11 | 73563760 | G/A |
| 11 | 73563917 | T/C |
| 11 | 73570606 | T/C |
| 11 | 73589110 | T/C |
| 11 | 73599475 | A/G |
| 11 | 73663301 | C/T |
| 11 | 73692993 | C/G |
| 11 | 73693001 | C/G |
| 11 | 73715484 | A/G |
| 11 | 73716158 | C/T |
| 11 | 73716390 | C/T |
| 11 | 73716580 | T/C |
| 15 | 26453002 | A/T |
| 15 | 27580836 | A/C |
| 18 | 35129344 | A/G |
| 23 | 43099394 | C/T |
| 27 | 638235 | A/G |
| 27 | 668811 | G/A |
| 27 | 687323 | A/C |
| 27 | 735281 | C/T |
| 27 | 736945 | A/G |
| 27 | 738242 | G/A |
| 27 | 738269 | T/G |
| 27 | 745156 | C/T |
| 27 | 7683337 | T/G |
| 27 | 7706463 | A/G |
| 27 | 7709080 | T/A |
| 27 | 7715004 | C/A |
| 33 | 20728877 | G/A |
| 36 | 16778015 | C/T |
